# Tulip-Screw Head Disjunction from Posterior C2 Fracture Fixation Instrumentation

**DOI:** 10.1155/2020/5824383

**Published:** 2020-02-24

**Authors:** Halle E. K. Burley, Darius S. Ansari, Alexander von Glinski, Ryan Goodmanson, Benjamin Schell, Jens R. Chapman, Rod J. Oskouian

**Affiliations:** ^1^Seattle Science Foundation, Seattle, Washington, USA; ^2^Western University of Health Sciences, College of Osteopathic Medicine of the Pacific-Northwest, 200 Mullins Dr., Lebanon OR 97355, USA; ^3^University of Illinois College of Medicine, 1835 W Polk St., Chicago, IL 60612, USA; ^4^Swedish Neuroscience Institute, Swedish Medical Center, Seattle, Washington, USA; ^5^Department of Trauma Surgery, BG University Hospital Bergmannsheil, Ruhr University Bochum, Bochum, Germany; ^6^Hansjörg Wyss Hip and Pelvic Center, Swedish Hospital, Seattle, Washington, USA

## Abstract

This report presents an unusual case of instrumentation failure after posterior fixation of a C2 fracture and reviews currently available treatment alternatives. The patient, a 53-year-old female, initially presented to the emergency department at an outside facility with acute alcohol intoxication and acute neck pain following a fall from a ladder. CT demonstrated bilateral C2 pars fractures and unstable posteroinferior displacement of the posterior elements. She underwent an emergent C2 open-reduction internal fixation (ORIF) at the outside facility with 3.5 mm polyaxial synapse pedicle screws (DePuy Synthes, Switzerland). There were no known complications and the patient was discharged. Two years after the index operation, cervical CT scan at a different facility revealed that although the fracture was fully healed, bilateral tulip caps had detached from the pedicle screw heads at C2. All implants were removed without postoperative complications. Industry review of alternate lag screws approved for the cervical spine demonstrated that there is not currently an ideal implant for fixation of C2 fractures without fusion. Cannulated trauma screws, which are low profile and would have avoided the instrumentation failure seen here, are not currently FDA approved for the cervical spine.

## 1. Introduction

Traumatic spondylolisthesis of the axis, also known as a Hangman's fracture, is a common injury following motor vehicle accidents [1–3]. Effendi and colleagues classified these injuries into three groups based on mechanism. Type I fractures occur with axial loading and hyperextension. Type II fractures are hyperextension injuries with rebound hyperflexion. Type III fractures are primarily flexion injuries with rebound extension [4]. Levine and Edwards modified the system by adding a type IIA, which represents flexion-distraction injuries. These combined forces produce a characteristic bilateral fracture of the pars interarticularis, sparing the odontoid, with or without associated anterolisthesis of the vertebral body [2, 5]. Affected patients may or may not present with neurological symptoms, and if the fracture is nondisplaced and stable, it can be treated nonoperatively with external immobilization [3, 5, 6]. Type I and II fractures generally fall into this category and can be treated conservatively with a collar. In cases of excessive angulation of C2 on C3, disruption of the C2-3 disc space, extensive displacement (<4-6 mm), or failure of external orthoses, surgical fixation and/or fusion is indicated [5, 6]. The typical operation for an isolated C2 fracture involves posterior reduction and fixation with pars screws [7, 8]. Further ventral C2-3 discectomy and fusion is performed for fractures that demonstrate sufficient instability to warrant more extensive fixation or in cases of neurological deficit. In cases where an anterior approach is contraindicated, the construct will be subject to excess stress, or bone quality is poor and posterior fusion to C1 or even the occiput may be indicated. The anterior approach is preferred because it preserves rotation at the atlantoaxial joint [1]. Presented here is an unusual case of bilateral tulip detachment from the screw heads two years status post C2 open-reduction internal fixation (ORIF) with polyaxial pedicle screws for a type I traumatic C2 fracture without known additional trauma.

## 2. Case Material

**Figure 1:**
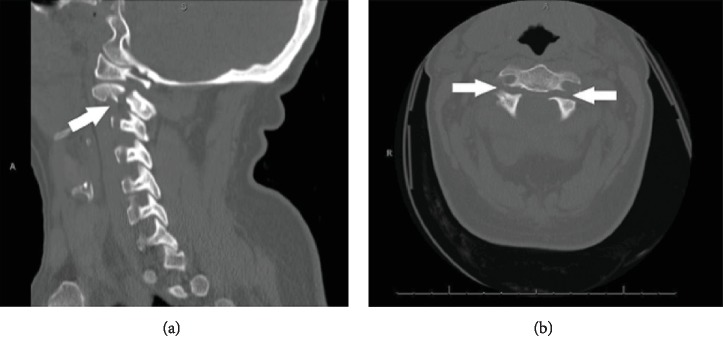
Fracture of C2 pedicles shown with white arrows. Sagittal view (a) and axial view (b).

A 53-year-old female presented to the emergency department of an outside facility with acute alcohol intoxication and acute neck pain following a fall from a ladder. Computed tomography (CT) imaging of her cervical spine demonstrated bilateral C2 pars fractures. There was no listhesis of the body of C2, but the posterior elements were displaced posteroinferiorly and determined to be unstable by the outside hospital's radiologist (Figure 1). Physical examination demonstrated intact cranial nerves II-XII, intact sensation, and full motor strength in all extremities. Given the displacement of the fracture and her physically active lifestyle, it was determined by her care team at the outside facility that surgical treatment was warranted. She underwent an emergent C2 open-reduction internal fixation (ORIF) at the outside facility. According to the operative report, the procedure was performed from a posterior approach via standard midline incision at C1-4 with the patient prone. Polyaxial 3.5 mm pedicle screws (SYNAPSE System, DePuy Synthes, Switzerland) were implanted bilaterally. There were no known complications and the patient was discharged home after the operation. It is unclear whether she followed up with her surgeon after the index operation, but she did participate in a course of physical therapy.

Eight months after the index operation at the outside facility, she presented to our clinic for reevaluation, complaining of posterior neck pain that had not improved since surgery. She was experiencing frequent headaches, new onset vertigo, and limited range of motion unresponsive to conservative management. A CT scan of the cervical spine revealed a healed C2 fracture with intact instrumentation. The patient was referred to further physical therapy with massage and scheduled for a repeat CT scan in 1 year.

She instead returned at 9 months with concerns of a firm, mobile bump at the posterior aspect of her neck accompanied by shooting pain radiating to the head. X-rays performed at this visit demonstrated stable C2 lateral mass screws with no other interval changes, and she continued conservative management at this time.

**Figure 2:**
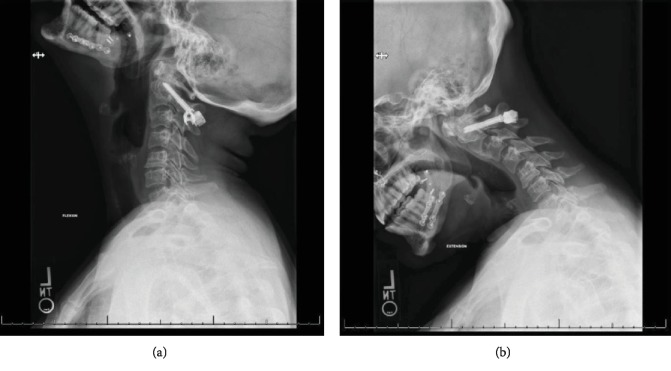
Radiographs of the cervical spine in extension (a) and flexion (b) display complete detachment of the tulip from the screw.

At her scheduled 2-year follow up, she continued to complain of a posterior neck mass and pain. A CT scan revealed that bilateral tulip caps had detached from the pedicle screw heads at C2. The patient's original fracture had healed, without any evidence of pseudarthrosis. Due to her discomfort, surgical removal of the instrumentation was recommended. One month prior to the planned instrumentation removal, flexion and extension radiographs of the cervical spine were obtained in order to visualize the overall bony anatomy and to assess the stability of the cervical spine (Figure 2). These images reaffirmed the findings of the previous CT, demonstrating detached bilateral screw heads with a stable, healed C2 fracture. No grossly mobile segments or listhesis was appreciated on these films.

**Figure 3:**
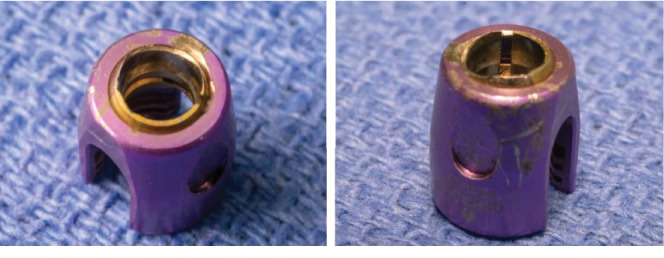
Erosion of the coupling portion of the tulip head from motion at the tulip-screw junction.

**Figure 4:**
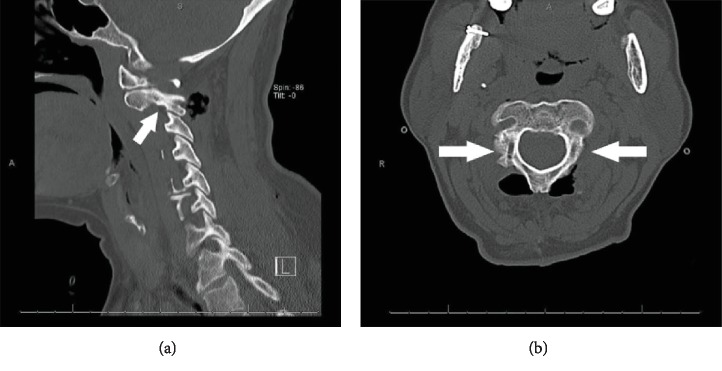
Healing of original C2 pedicle fractures shown with white arrows. Sagittal view (a) and axial view (b).

The patient underwent an elective instrumentation removal. She was positioned prone and the C2 level was exposed through a midline incision. The tulip heads were visibly disengaged from their respective screws and extensive metallosis was observed in the surrounding tissue. Upon inspection of the implant, there was erosion of the coupling portion of the tulip head (Figure 3). Once the tulips were removed, the pedicle screws were removed at C2 bilaterally and the incision was closed. Postoperative CT scan (Figure 4) demonstrated removal of instrumentation with complete healing of the original C2 fracture. The postoperative course was uncomplicated and she was discharged in stable condition.

## 3. Discussion

This case demonstrates a unique instance of instrumentation failure after successful osteosynthesis of a traumatic avulsion fracture of the neural arch of C2 in a physically active, otherwise healthy 52-year-old female. Instrumentation failures most commonly occur at points of maximum biomechanical stress. These can occur at the fracture lines or at the junction between multiple components, like the junction between the tulip head and pedicle screw head [9, 10]. In the absence of known trauma during the postoperative period, we hypothesize that the repetitive stress of the suboccipital muscle mass against the polyaxial screw when the patient extended her neck eroded the junction between the screw and polyaxial head due to repetitive cycling of the polyaxial head on the pedicle screw over time, causing it to dislodge. Alternately, the patient's extensive course of physical therapy and massage may have caused the tulip heads to dislodge. The prominence of the implants likely irritated the paraspinal muscles, causing the patient greater than usual postoperative muscle tension and discomfort. This would prompt her to undergo more intensive physical therapy and massage, thereby stressing the tulip-screw junction more than it was designed to withstand as an independent unit.

Prominent spinal instrumentation, while not necessarily problematic for the success of a fusion, can cause a considerable amount of discomfort for the patient postoperatively. For instance, in patients with prominent instrumentation following scoliotic deformity correction, skin irritation at the site of prominence is a potential cause of dissatisfaction and even reoperation [11]. Such complaints are less commonly reported in the cervical region [12]. More commonly, this is reported with instrumentation of the thoracolumbar and pelvic regions. The lower rate of complaints postcervical fusion is likely due to local soft tissue coverage and/or the infrequency of direct pressure to the cervical region in the course of daily activities [13]. In this clinical vignette, the prominence of the instrumentation likely caused irritation that prompted continued office visits, imaging, and therapy for the patient which could have led to the instrumentation failure.

The pedicle screws selected by the outside facility for this procedure, while unorthodox as a stand-alone unit, did serve their purpose of reducing the fracture. According to industry-provided information, the section of nonthreaded shaft adjacent to the screw head was originally designed to protect surrounding soft tissues. Although they were not intended to be used as lag screws, this feature incidentally resulted in effective reduction of the fracture fragments and produced a solid, bony fusion. However, despite the generally favorable outcome of the fixation technique, in retrospect, the use of a lag or positional screw without a tulip may have prevented the complications that this patient faced before ultimately undergoing an additional procedure.

The challenge, of course, is in the availability of such implants. Bilateral C2 arch fractures that are unstable enough to require surgical fixation but do not occur concomitantly with fractures to surrounding vertebrae are relatively rare, making the development of specialized implants for their repair not financially feasible [14]. The ideal screw for this type of repair would be a lag screw, not a polyaxial pedicle screw. Many orthopedic implant companies offer cervical spinal screws with fracture-reducing capabilities, but all come with a nonremoveable tulip head (Table 1). At this point in time, there is not an ideal, approved device on the market for posterior approach C2 arch fracture fixations not involving a fusion. This is likely why a screw with a superfluous, and later symptomatic, tulip head was used in this case. In typical cases of fracture fixation where a lag screw is used to create compression at the fracture site and bony union, 3.5-4.0 mm cortical- or cancellous-type trauma screws can be used with success [15, 16]. In theory and in practice, these could also be used to reduce and stabilize cervical fractures, but currently, they are not FDA approved for cervical spine use.

This case demonstrates that implants can still fail despite solid bone healing. Best practices would be to solve problems by using implants as they have been designed and intended. However, sometimes proper implants may not be available or exist to solve the problem in a way that is best for the patient. This case highlights the value of conservative use of instrumentation in surgery and the need for specialized implants for the fixation of isolated traumatic C2 fractures.

## Figures and Tables

**Table 1:** Currently available posterior cervical screws.

Company	Device/system	Modular	Removeable tulip	Partially threaded component	Other features
Stryker	OASYS	Yes	No	Yes	n/a
	YUKON	No	No	No	n/a

Medtronic	Infinity	Yes	No	No	n/a

Zimmer Biomet	Virage	No	No	Yes	Varying thread forms
	Lineum	No	No	Yes	Translational tulip

Globus	QUARTEX	No	No	Yes	n/a
	ELLIPSE	No	No	Yes	Nonthreaded locking cap

DePuy Synthes	SYNAPSE	No	No	Yes	n/a
	MOUNTAINEER	No	No	Yes	Laminoplasty-capable
